# Quantification of Uncoupled Spin Domains in Spin-Abundant Disordered Solids

**DOI:** 10.3390/ijms21113938

**Published:** 2020-05-30

**Authors:** Brennan J. Walder, Todd M. Alam

**Affiliations:** Sandia National Laboratories, Department of Organic Materials Science, Albuquerque, NM 87185, USA; tmalam@sandia.gov

**Keywords:** solid-state NMR, quantitative NMR, CPMG, layered carbon, carbon monofluoride, CF_x_, disordered solid

## Abstract

Materials often contain minor heterogeneous phases that are difficult to characterize yet nonetheless significantly influence important properties. Here we describe a solid-state NMR strategy for quantifying minor heterogenous sample regions containing dilute, essentially uncoupled nuclei in materials where the remaining nuclei experience heteronuclear dipolar couplings. NMR signals from the coupled nuclei are dephased while NMR signals from the uncoupled nuclei can be amplified by one or two orders of magnitude using Carr-Meiboom-Purcell-Gill (CPMG) acquisition. The signal amplification by CPMG can be estimated allowing the concentration of the uncoupled spin regions to be determined even when direct observation of the uncoupled spin NMR signal in a single pulse experiment would require an impractically long duration of signal averaging. We use this method to quantify residual graphitic carbon using ${}^{13}$C CPMG NMR in poly(carbon monofluoride) samples synthesized by direct fluorination of carbon from various sources. Our detection limit for graphitic carbon in these materials is better than 0.05 mol%. The accuracy of the method is discussed and comparisons to other methods are drawn.

## 1. Introduction

The microstructure and heterogeneity of materials exert significant influence on their macroscopic properties. The bioavailability of active pharmaceutical ingredients in drugs [1], deformation behavior of metallic glasses [2], progression of strength during cement hydration [3], and electrical properties of carbon composites in Li-ion battery electrodes [4] are just a few of many exemplifying cases. Characterization of microstructure is therefore an important part of establishing structure-property relationships, but doing so in a way that quantifies the number of distinct species present on a molecular basis is challenging. This is particularly so for disordered materials, where the quantitative information that can be obtained by X-ray diffraction (XRD) is limited. Methods such as X-ray photoelectron spectroscopy (XPS) and scanning electron microscopy/energy dispersive X-ray spectrometry (SEM/EDS) are capable of quantitative chemical analysis, but are usually limited to regions near particle surfaces. Furthermore, complicated sample topology and X-ray polarization effects make the acquisition of suitable reference data essential [5,6]. Without consideration of these effects, an order of magnitude error in quantification is possible.

Solid-state NMR spectroscopy with magic-angle spinning (MAS) is isotope specific, can resolve distinct functional groups, and is intrinsically quantitative as the intensity of a directly excited NMR signal is proportional to the number of nuclear spins in the sample. Solid-state NMR, however, is an insensitive method, and NMR signals from spins with relatively low concentrations can be difficult to observe. To overcome this it is conventional, when possible, to transfer nuclear polarization from a pool of abundant, strongly magnetic nuclei such as ${}^{1}$H or ${}^{19}$F to dilute, weakly magnetic nuclei such as ${}^{13}$C (1.11% natural abundance) for detection using the method of cross-polarization (CP) [7,8]. General strategies for hyperpolarization of nuclei in solids under MAS using dynamic nuclear polarization have also emerged in recent years [9,10]. While these methods can deliver tremendous sensitivity and resolution improvements, the efficiency of polarization transfer and relative signal enhancements depend on numerous component-specific factors, invalidating straightforward quantitative analysis of signal intensities.

Methods to restore the quantitative aspect while retaining the benefits of CP have been developed for materials where abundant nuclei are accessible to all phases of interest, typically organic matter [11,12,13]. Nevertheless, when CP enhanced polarization is inaccessible to the phases of interest due to practically nonexistent heteronuclear couplings between the dilute spins and the abundant spins, for example graphitic domains in organic solids, one must fall back on comparatively insensitive direct excitation methods which leverage the equilibrium polarization. For disordered solids, where distributions in the chemical shift lead to significant inhomogeneous line broadening, this is a severe practical limitation. It is often the case, however, that the transverse relaxation time, $T_{2}$, of dilute nuclei lacking significant coupling to abundant spins are many orders of magnitude larger than the duration of their NMR signal envelope, $T_{2}^{*}$. The relatively slow transverse relaxation is exploited in the Carr-Purcell-Meiboom-Gill (CPMG) experiment [14] for solids undergoing MAS [15,16,17], in which a series of refocussing pulses are applied synchronously with the sample rotation to refocus inhomogeneous (e.g., chemical shift) interactions and form a train of spin echoes. The echo train allows one to accumulate copies of the original signal in quick succession prior to complete spin relaxation, enhancing the sensitivity of the experiment. By suitable analysis, the component intensity prior to any relaxation can be determined, allowing CPMG data to be interpreted quantitatively. Demonstrations of quantitative analysis by CPMG include ${}^{29}$Si NMR of oxide glasses, mesoporous silica, and ${}^{119}$Sn NMR of zeolites [18,19,20]. Other quantitative solid-state NMR experiments exploiting $T_{2}>T_{2}^{*}$ regimes to enhance sensitivity include the flip-back based uniform Driven Equilibrium Fourier Transform (UDEFT) experiment [21] and phase incremented echo train acquisition (PIETA) [22]. The PIETA experiment, related to CPMG, can yield quantitative spectra that also correlates the evolution of spin interactions not refocussed by the echo train pulses such as *J* couplings. This additional correlation enhances spectral information and has been used to quantify structural distributions in silica glass [23].

In this work we describe a CPMG-based NMR experiment that can be used to enhance the NMR signals from domains containing essentially uncoupled dilute nuclei relative to NMR signals from domains where the dilute nuclei can experience residual (homogeneous) evolution under the through-space heteronuclear dipolar coupling to abundant nuclei that remains despite the averaging effect of MAS [24]. The former nuclei possess very long $T_{2}$ and form a train containing many echoes. NMR signals from the latter type of nuclei are recorded in the free induction decay (FID) under heteronuclear decoupling applied to the abundant spins and then are suppressed in the echo train by reinstating their residual dipolar interactions with the abundant nuclei. The NMR signals recorded in the echo train are used to reconstruct an amplified NMR signal corresponding to the uncoupled nuclei, providing a powerful means of contrast by way of selective, sensitivity enhanced observation of the uncoupled spins. We show that the degree of amplification can be calculated allowing the concentration of uncoupled spins in the sample to be determined. With this method we carry out a quantitative analysis of residual graphite in bulk poly(carbon monofluoride), (CF)${}_{n}$, a conversion cathode material used in lithium primary batteries [25] whose electrical conductivity is affected by the concentration of graphitic sp${}^{2}$ carbon [26]. We show that the mole fraction of residual graphitic carbon can be accurately determined down to a limit of 0.05 mol% and discuss factors that limit the accuracy of the measurement.

## 2. Results

### 2.1. Characteristics of Poly(Carbon Monofluoride) Samples

Our (CF)${}_{n}$ samples were synthesized by direct fluorination of carbon by F${}_{2}$ gas at high temperature. When run to completion the reaction yields a bulk consisting of nanometer-sized platelets having a fluorographene structure [27].

**Figure 1:**
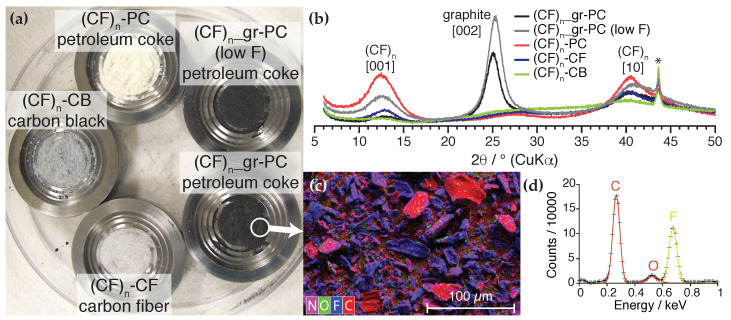
Characteristics of bulk poly(carbon monofluoride) samples. (**a**) Photographs of the five samples overlaid by their names and their carbon sources; (**b**) X-ray diffraction spectra for the samples; (**c**) SEM/EDS map of (CF)${}_{n}$_gr-PC; (**d**) photon counts for the EDS mapping shown in (**c**). The asterisk (*) in (**b**) is an artifact from the sample holder. The elemental color coding for the SEM/EDS mapping is given in the lower left corner of (**c**).

We investigate a series of commercial (CF)${}_{n}$ samples made using different carbon sources: petroleum coke (PC), carbon black (CB), or carbon fiber (CF). Photographs of these samples are shown in Figure 1a. The three (CF)${}_{n}$ powders on the left, one from each source, are “fully fluorinated“, whereas the two black powders on the right are “sub-fluorinated“ (CF)${}_{n}$, resulting from incomplete fluorination of petroleum coke. The sub-fluorinated samples contain a significant fraction of graphitic carbon (gr) that is responsible for their black appearance. The three fully fluorinated samples, on the other hand, appear as varying shades of gray. (CF)${}_{n}$-PC is nearly white, whereas (CF)${}_{n}$-CF and (CF)${}_{n}$-CB are progressively darker. The powder XRD (pXRD) spectra of the three (CF)${}_{n}$ samples, shown in Figure 1b, exhibit relatively broad peaks due to disorder. Peaks near 2θ = 13${}^{\circ }$ and 41${}^{\circ }$ correspond respectively to the [001] and [10] reflections of (CF)${}_{n}$ [28,29], strongest in (CF)${}_{n}$-PC and weakest for (CF)${}_{n}$-CB. The spectra of the sub-fluorinated (CF)${}_{n}$_gr-PC powders are dominated by a reflection near 2θ = 25${}^{\circ }$ that is best explained by the [002] reflection of turbostratic graphite with a slightly enhanced average interlayer spacing [30].

SEM/EDS, shown for the sub-fluorinated (CF)${}_{n}$_gr-PC sample with a higher overall fluorine content (hereafter just “(CF)${}_{n}$_gr-PC“) in Figure 1c, reveals the nature of the heterogeneity. Fluorine is detected on the surface of every particle, but large fluorine-free regions are observed for some particles, particularly the larger ones. This suggests that the graphitic carbon occurs as residual domains embedded within fully fluorinated (CF)${}_{n}$, consistent with incomplete fluorination of the carbon source. A graph of the photon counts corresponding to the EDS mapping in Figure 1c is given in Figure 1d. Peaks corresponding to carbon, oxygen, and fluorine are observed in the sub-keV region. Oxygen results from the adhesive backing used in the EDS analysis and intercalated O${}_{2}$ gas [31]. A certain degree of carbon also results from the adhesive. Assuming all fluorine counts correspond to fluoromethanetriyl (>CF-) functional groups, a rudimentary quantitative analysis based upon the peak areas suggest a graphitic carbon fraction of 30 mol%.

### 2.2. Uncoupled ${}^{13}$C Enhanced CPMG MAS NMR

**Figure 2:**
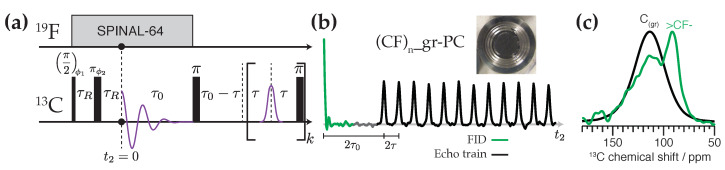
Direct excitation CPMG NMR for enhancement of ${}^{13}$C in spin-sparse domains: (**a**) pulse sequence, (**b**) experimental ${}^{13}$C MAS NMR time domain signal (real part) from (CF)${}_{n}$_gr-PC up to the twelfth echo (of thirty thousand), showing the FID (green) and echo train (black), (**c**) frequency domain ${}^{13}$C MAS NMR spectrum of (CF)${}_{n}$_gr-PC using the FID (green) or the matched reconstructed echo (black). For the experimental data k=30,000, $\tau_{R}=60\;\mu$s, $\tau_{0}=2.4$ ms, and τ=0.6 ms. The $\tau_{R}$, $\tau_{0}$, and τ intervals are measured with respect to the dashed lines or the center of the π pulses. Nested cycling of $\phi_{1}$ and $\phi_{2}$ selects two $p_{I}$ symmetry pathways [32] prior to CPMG: {0→+1→−1} and {0→−1→+1}. Both time and frequency domain spectra have been apodized to improve presentation. The pulse sequence is available in the Supplementary Materials.

The NMR pulse sequence we introduce for quantifying spin-sparse domains in spin-abundant solids is shown in Figure 2a. The initial 2$\tau_{R}$ interval, where $\tau_{R}$ is the rotor period, generates a spin echo at $t_{2}=0$. The FID is defined as the NMR signal decay off of this point. Because of the short echo shift, the FID can be acquired without distortions due to receiver dead time. Though transverse relaxation does occur during the initial 2$\tau_{R}$ interval, its duration is very short relative to the $T_{2}$ values of not only the graphitic ${}^{13}$C but also the fluorinated ${}^{13}$C when high-power ${}^{19}$F decoupling is applied. Therefore inaccuracy due to this prior relaxation can be neglected. The ${}^{19}$F decoupling is continued during acquisition of the FID to improve resolution. The duration $\tau_{0}$ is selected based on the spectral resolution desired in the FID, with $\tau_{0}$ much longer than $T_{2}^{*}$(FID) improving resolution but impairing the signal-to-noise (S/N) ratio of the FID. The ${}^{19}$F decoupling is terminated just prior to application of the first ${}^{13}$C pulse of the CPMG train. This reinstates homogeneous ${}^{13}$C spin evolution under the residual ${}^{19}$F dipolar couplings, preventing the NMR signal of the ${}^{19}$F-coupled ${}^{13}$C from refocussing when $t_{2}=2\tau_{0}$. Because the generally narrower ${}^{19}$F-coupled ${}^{13}$C NMR signals are suppressed in the echo train, $T_{2}^{*}$(CPMG) ≤$T_{2}^{*}$(FID), allowing us to set $\tau \leq \tau_{0}$ without truncation of the echo signals. Both intervals must remain a multiple of $\tau_{R}$. The data sampling interval should also be set so that an integer number of points are sampled during the $\tau_{0}$ and τ intervals, which facilitates echo summation. A total of *k* echoes are collected. The top of the $j^{\mathrm{th}}$ echo (1≤j≤k) is defined to occur at $t_{2}^{\left(j\right)}=2\left(j-1\right)\tau +2\tau_{0}$.

The decay of the intensity of the refocussed echoes along the CPMG echo train, E(j), is governed by transverse ($T_{2}$) as well as longitudinal ($T_{1}$) relaxation processes, the latter of which are blended in over the course of the echo train by pulse imperfections [33,34], resulting in a multiexponential decay for E(j). Intrinsic multiexponential NMR relaxation is commonly observed for dilute nuclei in solids [35,36,37,38]. It is common to parameterize complicated multiexponential behavior using a stretched exponential function [18,37,38,39], and so for E(j) we write

(1) $$E\left(j\right)=\exp\left[-{\left(\frac{2\left(j-1\right)\tau +2\tau_{0}}{T_{\mathrm{CPMG}}}\right)}^{\beta }\right],$$

where $T_{\mathrm{CPMG}}$ is the characteristic time constant for the decay and β is the dimensionless stretching exponent. The intensity of the $j^{\mathrm{th}}$ echo top in the train, occurring at $t_{2}^{\left(j\right)}$, is proportional to E(j). In our samples $T_{\mathrm{CPMG}}$ for the graphitic domains can exceed 10 s, permitting the generation of tens of thousands of ${}^{13}$C echoes during a single acquisition cycle. These signals manifest sharply in the time domain, as seen in Figure 2b. For the fluorinated domains, $T_{\mathrm{CPMG}}$ is orders of magnitude shorter and our pulse sequence dephases these contributions prior to the first echo. This is demonstrated in Figure 2c, in which the FID signal from (CF)${}_{n}$_gr-PC yields a spectrum that contains both a broad signal (fwhm = 35 ppm) between roughly 70 ppm and 150 ppm and a sharper signal (fwhm ≈ 15 ppm) around 90 ppm. The latter resonance is assigned to fluorinated carbon. The underlying broad signal is due to the substantial amount of graphitic carbon in this sample. In contrast, when the thirty thousand echoes from the CPMG train are used to reconstruct the signal enhanced ${}^{13}$C NMR spectrum, only the long-lived graphitic ${}^{13}$C signal can be discerned.

### 2.3. Signal Amplification by the Uncoupled Spin Enhanced CPMG NMR Experiment

The reconstructed echo signal is created by weighted summation of the *k* echoes by a procedure which is formalized in Appendix A. The result with which we are concerned is the NMR signal amplification of the uncoupled spin regions by CPMG. We define the gain function, G(k), which expresses the increase in S/N of the NMR signal component in the weighted reconstructed echo due to the uncoupled spins (in this case graphitic ${}^{13}$C) over the S/N of the same NMR signal component in the FID. It can be calculated from E(j) and the mathematically exact weighting function, h(j), according to

(2) $$G\left(k\right)=2\sqrt{\frac{N_{0}}{N_{W}}}\frac{\sum_{j=l+1}^{k}h\left(j\right)E\left(j\right)}{\sqrt{\sum_{j=l+1}^{k}{\left[h(j)\right]}^{2}}}.$$

The numerator is the amplification of the NMR signal in the weighted reconstructed echo, whereas the denominator gives the amplification of the noise. The number of points in the FID and weighted reconstructed echo are $N_{0}$ and $N_{W}$, respectively, and the factor $\sqrt{N_{0}/N_{W}}$ accounts for the different extent of time domain noise included in each signal. When the weighting function is set equal to the envelope function, h(j)=E(j), we obtain the matched gain function, $G_{M}\left(k\right)$:

(3) $$G_{M}\left(k\right)=2\sqrt{\frac{N_{0}}{N_{W}}\sum_{j=l+1}^{k}{\left[E(j)\right]}^{2}}.$$

It can be shown that this choice of h(j) maximizes G(k) for all values of *k*, in line with the principles of matched filtering [40,41].

The gain function is easily related to the practical sensitivity of the experiment, S(k), which compares the sensitivity of the CPMG NMR experiment to the standard acquisition of a single FID by direct excitation for the component being enhanced. This is done by accounting for the longer acquisition period in the CPMG experiment,

(4) $$S\left(k\right)=\sqrt{\frac{b+j_{0}}{b+2k+2j_{0}-1}}G\left(k\right).$$

Here we define $j_{0}=\tau_{0}/\tau$ as well as $b=\tau_{\mathrm{rd}}/\tau$, where the delay between scans, $\tau_{\mathrm{rd}}$, is assumed to be determined by the time required for full longitudinal relaxation of the spin ensemble. We consider the practical experimental sensitivity based on the unweighted gain $G_{U}\left(k\right)$, where h(j)=1∀j, and the matched gain function $G_{M}\left(k\right)$, defined in Equation (3). Assuming E(j) obeys Equation (1), these gain functions are given by

(5) $$\begin{array}{cc} G_{U}\left(k\right) & =\sqrt{\frac{2j_{0}}{k-l}}\sum_{j=l+1}^{k}\exp\left[-{\left(\frac{2(j+j_{0}-1)}{a}\right)}^{\beta }\right], \end{array}$$

(6) $$\begin{array}{cc} G_{M}\left(k\right) & =\sqrt{2j_{0}\sum_{j=l+1}^{k}\exp\left[-2{\left(\frac{2(j+j_{0}-1)}{a}\right)}^{\beta }\right]}, \end{array}$$

where we have defined the dimensionless decay parameter $a=T_{\mathrm{CPMG}}/\tau$. The experimental sensitivity functions $S_{U}\left(k\right)$ and $S_{M}\left(k\right)$ are obtained by substituting Equations (5) and (6) into Equation (4), respectively.

**Figure 3:**
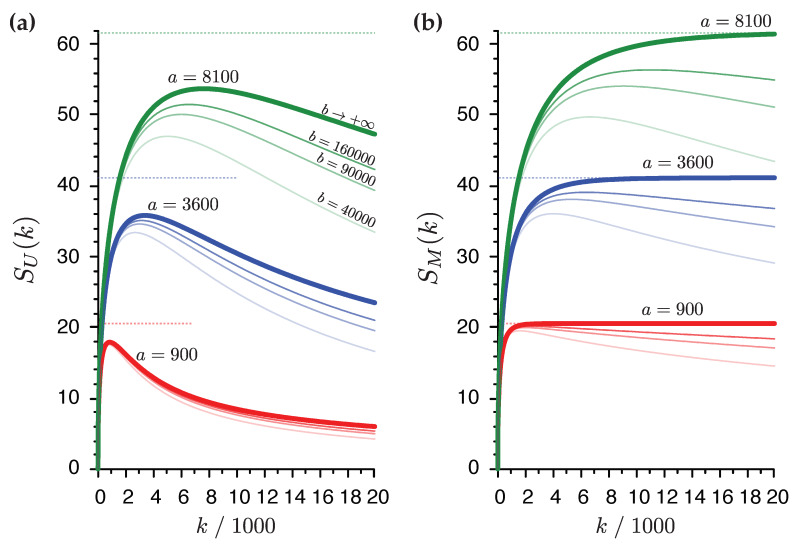
Sensitivity enhancement by the CPMG NMR experiment plotted against the number of echoes acquired, *k*, using (**a**) the unweighted sensitivity function $S_{U}\left(k\right)$ (**b**) the matched sensitivity $S_{M}\left(k\right)$. Three families are distinguished on the basis of the value of $a=T_{\mathrm{CPMG}}/\tau$: a=900 (red), a=3600 (blue), a=8100 (green). Four branches are plotted for each family corresponding to different values of $b=\tau_{\mathrm{rd}}/\tau$: b=40,000, b=90,000, b=160,000, and b→+∞, in order of increasing opacity. The lines in bold stroke correspond to Equations (5) or (6). The dashed lines indicate the asymptotic value of $G_{M}\left(k\right)$ for the corresponding value of *a*. The values of $\beta =\frac{2}{3}$, $j_{0}=1$, and l=0 are common to all curves plotted.

The sensitivity functions $S_{U}\left(k\right)$ and $S_{M}\left(k\right)$ are plotted in Figure 3 as a function of the number of echoes collected, *k*. All curves exhibit a steep initial rise where $S\propto \sqrt{k}$ and the values of their maxima are roughly proportional to $\sqrt{a}$. While $S_{U}\left(k\right)$ attains a maximum value for k≲a, $S_{M}\left(k\right)$ exhibits much flatter maxima which occur when k≳a for finite *b*. As expected, the matched sensitivities exceed that of the unweighted sensitivities, for all values of *k*. In particular, the decay in the region k≫a of $S_{M}\left(k\right)$ is never worse than $k^{-1/2}$, whereas for $S_{U}\left(k\right)$ it is no better than $k^{-1/2}$. These properties of $S_{U}\left(k\right)$ at large *k* are consequences of unfavorable accumulation of noise in the summation after the NMR signal contained in each echo has largely decayed. We also see that the sensitivity increases as *b* increases, particularly at large *k*, implying the time it takes to collect additional echoes is less penalizing the larger the wait between scans.

In the limit b→+∞, the corresponding gain functions $G_{U}\left(k\right)$ and $G_{M}\left(k\right)$ are returned. For all experimentally sensible values of β, $G_{M}\left(k\right)$ approaches a limiting value as k→+∞. This value represents the maximum signal enhancement that can be achieved theoretically for a given value of *a* and is roughly equal to $\frac{2}{3}\sqrt{a}$. Moreover, for large *k* the gain is relatively independent of the number of echoes. These useful properties are not shared by the unweighted $G_{U}\left(k\right)$. Thus, efforts to match the envelope function should *always* be made.

The dependence of $S_{M}\left(k\right)$ on β and *l* is illustrated in Figures S1 and S2, respectively.

### 2.4. Quantification of Graphitic Carbon in Poly(Carbon Monofluoride)

**Figure 4:**
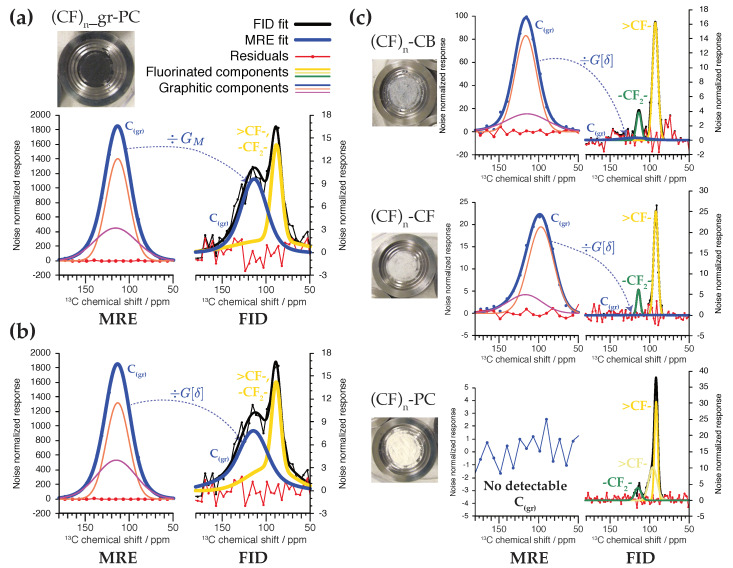
Quantification of graphitic ${}^{13}$C MAS NMR signals by downscaling the noise normalized $\left(\sigma \left(N_{0}\right)=\sigma \left(N_{W}\right)=1\right)$ matched reconstructed echo onto the FID. (**a**) Scaling of the MRE onto the FID of (CF)${}_{n}$_gr-PC using a fixed gain of $G_{M}=192.2$. (**b**) Scaling of the MRE using a chemical shift dependent gain function G[δ] determined from analysis of differential $T_{\mathrm{CPMG}}$ relaxation. (**c**) Comparison of the FID and MRE signals for the fully fluorinated (CF)${}_{n}$ samples.

Figure 4 shows how the NMR signal in the matched reconstructed echo (MRE), $S_{W}\left(\nu \right)$, is used in the determination of the amount of graphitic ${}^{13}$C NMR signal present in the FID. To permit comparison by the gain functions, both the MRE and FID are normalized such that the standard deviations of the noise level for the FID, $\sigma \left(N_{0}\right)$, and MRE, $\sigma \left(N_{W}\right)$, are set equal to unity. For (CF)${}_{n}$_gr-PC in Figure 4a, ${}^{13}$C NMR signals for both graphitic and fluorinated carbon can be discerned in the FID, though they are not well-resolved. We model the graphitic NMR signals using two normal distributions, as modeling by a single analytic distribution yields relatively poor fits. Similarly, the fluorinated NMR signals are modeled using at least two normal distributions. Detailed interpretation of such signal decomposition is unnecessary for our purposes.

Parameters for the matching h(j) were chosen by analyzing the integrated intensities of (CF)${}_{n}$_gr-PC echoes in the frequency domain to characterize E(j), yielding $T_{\mathrm{CPMG}}=\left(12.769\pm 0.048\right)$ s and β=0.571±0.002, as described at length in Section 4 of the Supplementary Material. From this and the experimental parameters we calculate the gain $G_{M}\left(30000\right)\equiv G_{M}=192.2$ using Equation (6). When $S_{W}\left(\nu \right)$ is downscaled using this $G_{M}$ value, we see in Figure 4a that its intensity maps well onto the graphitic ${}^{13}$C NMR signal observed in the FID. With this mapping as a constraint, fitting for the graphitic and fluorinated ${}^{13}$C NMR signal components (areas) for (CF)${}_{n}$_gr-PC simultaneously finds that the mole fraction of graphitic carbon in the sample is $x_{\mathrm{gr}}=\left(48.5\pm 3.6\right)$ mol%. The broad fluorinated component on which the narrower >CF- signature rests is a real feature that can be observed in the ${}^{13}$C CP NMR spectrum of (CF)${}_{n}$_gr-PC, which is shown in Figure S3.

The residuals shown in Figure 4a of the NMR signal in the FID are not significantly different from noise (rms = 1.23), suggesting the model is a suitable description of the data. Nevertheless, significant variations of E(j) as a function of chemical shift δ can be measured (E(j)[δ]), as shown in Figure S4a. Thus, we present in Figure 4b an analysis of (CF)${}_{n}$_gr-PC using a chemical shift dependent downscaling, G(30000)[δ]≡G[δ]. This decreases the contribution of $S_{W}\left(\nu \right)$ to the FID near the middle of the spectrum but increases its contribution near the edges. Consequently, the quantitative result for $x_{\mathrm{gr}}$ does not significantly change, and we now determine $x_{\mathrm{gr}}=\left(45.7\pm 2.7\right)$ mol%. The residuals of the NMR signal in the FID for this model improve slightly to an rms value of 1.08, statistically and visually indistinguishable from noise.

With these insights at hand we measure $x_{\mathrm{gr}}$ for the “fully fluorinated” samples, which ideally do not contain graphitic domains. For these samples the fluorinated carbon signals get progressively narrower; thus, to avoid truncation of these signals in the FID, $j_{0}$ is progressively increased, which drives a corresponding increase in $G_{M}\left[\delta \right]$ (and $G_{M}$), as shown in Figure S4b. The result of the fits are shown in Figure 4c and summarized in Table 1, assuming the parameters describing E(j)[δ] for these samples are related to those measured for (CF)${}_{n}$_gr-PC. Only (CF)${}_{n}$-PC yields no detectable graphitic ${}^{13}$C NMR signal. For (CF)${}_{n}$-CB and (CF)${}_{n}$-CF graphitic ${}^{13}$C can be detected in the MRE, but constitutes such a small contribution to the FID that *its existence cannot be inferred without the aid of the CPMG enhanced signal*. For (CF)${}_{n}$-PC we estimate an upper bound to $x_{\mathrm{gr}}$ based upon the lowest value that would yield discernible MRE signal given the expected $G_{M}=303.7$. This establishes $x_{\mathrm{gr}}=0.05$ mol% as the practical limit of detection.

## 3. Discussion

Our results for $x_{\mathrm{gr}}$ correlate well with the visual grayness of the samples. About half the carbon by mole is graphitic in the black (CF)${}_{n}$_gr-PC sample. Uncoupled ${}^{13}$C enhanced CPMG NMR easily detects graphitic carbon in (CF)${}_{n}$-CB and (CF)${}_{n}$-CF, whereas pXRD fails to register any graphite signature whatsoever, as seen in Figure 1b. It is likely that the residual graphite domains are too small for detectable diffraction at such high levels of fluorination. Similarly, the EDS mapping is unable to resolve different types of carbon. Our initial analysis of Figure 1d assumed all fluorine counts originated from fluoromethanetriyl groups, giving $x_{\mathrm{gr}}=30$ mol%, significantly less than the NMR results. This is to be expected from a standardless analysis subject to error by adhesive background signals, topological effects, and imperfect knowledge about secondary functional groups such as difluoromethylene (-CF${}_{2}$-) [5]. XPS, being sensitive to the atomic hybridization, would be expected to resolve the graphitic carbon from the fluorinated bulk, but given the vast size distribution and heterogeneity of the (CF)${}_{n}$ aggregates, as exemplified in the SEM of Figure 1c, such an analysis would also be subject to inaccuracies [6].

For graphite concentrations on the order of 1 mol%, it becomes difficult to estimate $T_{\mathrm{CPMG}}$ precisely. To circumvent this, our analysis assumes that the CPMG envelope function is described by the same parameters for all samples, which may be a significant source of systematic error affecting the accuracy of the results presented in Table 1. Figure S5 and Table S2 show that for (CF)${}_{n}$-CB a value of $T_{\mathrm{CPMG}}=\left(13.9\pm 0.8\right)$ s is obtained from directly fitting the intensities of the integrated signal region as a function of echo count, not significantly different than the value of $T_{\mathrm{CPMG}}=\left(12.77\pm 0.05\right)$ s found for (CF)${}_{n}$_gr-PC. This approach fails for (CF)${}_{n}$-CF unless β is constrained, giving the result $T_{\mathrm{CPMG}}=\left(26.5\pm 3.8\right)$ s for β=0.57. With this result Equation (2), with h(j) appropriate to (CF)${}_{n}$_gr-PC (≡G), now yields G=351.7, nearly 30% larger than the $G_{M}=271.7$ used in our analysis. Despite this significant difference, using this new value of *G* yields $x_{\mathrm{gr}}=\left(0.54\pm 0.12\right)$ mol%, the same as the corresponding value given in Table 1 within error. Note that if the gain were (unjustifiably) augmented by 30% in the analysis of (CF)${}_{n}$_gr-PC we would have obtained $x_{\mathrm{gr}}=\left(38.5\pm 2.9\right)$ mol%, a difference which is more significant. This shows that the level of systematic error incurred by inaccuracies in estimating $T_{\mathrm{CPMG}}$, β, and, by extension, $G_{M}$, depends on the intensity of signal component being enhanced by CPMG. When the long-lived signal component of interest is small relative to the other signals in the FID – that is, when our method is most useful – the systematic error is unlikely to exceed the random error resulting from uncertainties in the fit.

This also explains why the results obtained using the chemical shift dependent gain factor, G[δ], are statistically indistinguishable from the results using the integrated $G_{M}$ value despite strong variations measured across the line shape, as shown in Figure S4. For disordered solids it is common to measure relaxation that depends on the isotropic chemical shift and the challenges it poses for quantitative analysis by CPMG NMR was discussed by Malfait and Halter [18], who argued that accounting for the chemical shift dependence is crucial for accurate quantification. This is true when the relative intensities of different components in the echo train are to be compared, as in previous instances of quantitative NMR by CPMG [18,19,20]. In our case we do observe narrowing of the graphitic ${}^{13}$C NMR line shape as *j* grows large as well as a curious change in chemical shift for $S_{W}\left(\nu \right)$ of (CF)${}_{n}$-CF relative to (CF)${}_{n}$-CF and (CF)${}_{n}$_gr-PC (about -15 ppm). Should interpretation of such observations fall within the scope of this work the chemical shift dependence of *G* would be an important consideration. For the purpose of accurately quantifying $x_{\mathrm{gr}}$, however, we find that use of the averaged $G_{M}$ value measured on a reference sample is sufficient.

A potentially large source of error arises from imprecise estimation of the noise level used in separate normalization the signal intensities, especially since $N_{0}$ and $N_{W}$ are likely to be small in most practical cases. This source of error is eliminated if Equation (A6) is used to compare intensities. If desired, accurate noise normalization can still be established by phasing a 2D dataset of appended echo spectra such that the imaginary channel contains 2kN samples of independent noise, 2N for each properly phased echo (when *N* points are recorded over the period τ). From the 2kN samples the noise level of the $j^{\mathrm{th}}$ echo, $\sigma \left(N_{j}\right)$, can be calculated to a high degree of precision and set to unity. Scaling of $\sigma \left(N_{W}\right)$ to unity follows from the rooted summation factor of Equation (A11) and the scaling of $\sigma \left(N_{0}\right)$ to unity follows from the *Q* and rooted *N* factors. This was the protocol used in establishing the response scales in Figure 4.

Partial destruction of NMR signal due to rapid relaxation of ${}^{13}$C nuclei induced by proximity to radical defects (“bleaching”) must also be considered. Unpaired electrons occur as localized aromatic π-radical defects in the graphitic domains [42] and as dangling C–C bonds in the fluorinated domains [43]. Given the synthetic conditions commonly used in direct fluorination of graphite, these should manifest in similar concentrations on the order of $10^{19}$ spins/g within their respective domains [42,44]. This concentration is low enough that any bleaching is minimal, and because both domains are affected to a similar degree, our analysis of these (CF)${}_{n}$ samples are not led into inaccuracy by the neglect of radicals.

Note ${}^{13}$C contained within any interfacial sp${}^{2}$ carbon at the immediate boundary of a graphitic and fluorinated region of (CF)${}_{n}$ or within aromatic point defects may experience enough spin evolution under the residual couplings to ${}^{19}$F for its NMR signal to be dephased prior to $t_{2}=2\tau_{0}$. Such carbon would be accounted as fluorinated by our method. For meaningful heterogeneity of the material to exist, however, the distinct domains must be of a size that renders the relative concentration of interfacial carbon low enough that the systematic error by this way of accounting can be neglected.

Due to the extremely long $T_{1}$ and $T_{2}$ of essentially uncoupled nuclei, our technique is capable of delivering tremendous sensitivity enhancements. In order to directly observe the graphitic ${}^{13}$C NMR signal in the FID of (CF)${}_{n}$-CF ($x_{\mathrm{gr}}=0.7$ mol%) with a maximum S/N = 3 while preserving resolution of the narrow fluorinated carbon signals, the experiment would need to be run nearly 900 times as long – just over 3 years. Our method yielded a graphitic ${}^{13}$C NMR signature with a maximum S/N > 20 in just over 30 hours. Sensitive and quantitative detection of residual graphitic domains in fluorinated carbon by NMR is not currently feasible without CPMG. The improvement in contrast provided by CPMG enhancement of uncoupled spins should also benefit, for example, ${}^{29}$Si NMR investigations into the hydration of cement containing disordered slags [45] and tracking the quantity of carbon fiber produced from the pyrolysis of biopolymers such as lignin by ${}^{13}$C NMR [46].

## 4. Materials and Methods

Poly(carbon monoflouride) samples were obtained commercially from Advance Research Chemicals, Inc (Catoosa, OK). For each sample a roughly 5 mm long zone was centered into a 2.5 mm zirconia rotor, buffered with powdered NaCl, and closed with vespel caps.

NMR experiments were carried out at 9.4 T on a Bruker Ascend 400WB system using a 2.5 mm HF/X CP MAS probe and an Avance III HD spectrometer. The standard ${}^{13}$C reference was determined externally by referencing the ${}^{19}$F resonance of NH${}_{4}$CF${}_{3}$COO (=−72.0 ppm with respect to CFCl${}_{3}$) and scaling by the appropriate reference frequency ratio [47]. Hard ${}^{13}$C pulses of 114 kHz rf amplitude were used. SPINAL-64 with a pulse element of 4.7 μs and an rf amplitude of 120 kHz was used for ${}^{19}$F decoupling [48]. Samples were spun in compressed air in the presence of an auxiliary flow of 1500 L/h and 300.0 K to regulate temperature. Unless otherwise noted, the MAS rate was 16 23bevelledtrue kHz ($\tau_{R}=60\;\mu$s), stable to within 2 Hz. At this MAS rate the data sampling rate, 2*DW, was set to 60μs to fold weak ${}^{13}$C spinning sidebands onto the centerband, simplifying quantitative analysis. An integer number of complex points per echo were collected corresponding to N=10. For the FID, $j_{0}=4,6,8,\;\mathrm{and}\;10$ for (CF)${}_{n}$_gr-PC, (CF)${}_{n}$-CB, (CF)${}_{n}$-CF, and (CF)${}_{n}$-PC, respectively. The respective number of total scans (total experiment time) for each sample was 296 (62.16 h), 224 (47.04 h, acquired in two consecutive sessions with signals added together during processing), 144 (30.24 h), and 432 (90.72 h). Slightly more than 30000 echoes were set to be acquired as signal from the last several echoes were lost internally to digital filtering by the spectrometer electronics.

Direct excitation is quantitative when the ensemble of spins being observed is at thermal equilibrium prior to each scan. As shown in Figure S6, we ensure full relaxation by using a recycle delay of $\tau_{\mathrm{rd}}=12$ min. While this does incur a sensitivity penalty which can be avoided by correcting the measured signal intensities for the partial relaxation of the ensemble [49], we opted to use the longer $\tau_{\mathrm{rd}}$ to avoid introducing additional measurement-dependent corrections.

NMR signal processing was carried out using *RMN*, version 1.8.6 [50], which uses the dFT normalization convention $Q\left(N\right)=1/\sqrt{N}$. Uncorrelated noise floors were established for both FID and MRE signals based upon Equation (A11) such that the enhancement of the graphitic signal by CPMG is given by Equation (2). A stretched exponential weighting function $h\left(j\right)=\exp[-{\left(2\left(j+j_{0}-1\right)/a^{\prime }\right)}^{\beta^{\prime }}]$ with parameters $a^{\prime }=21281.5$ and $\beta^{\prime }=0.571097$, corresponding to the best fit envelope function measured by the integrated (CF)${}_{n}$_gr-PC echo intensities, was used unless otherwise specified.

Scripts to analyze the line shapes were written for *gnuplot*, version 5.2.8, and are provided in the Supplementary Materials. All NMR signal line shapes were decomposed into multiple overlapping normal distributions. The FID and MRE signals were fit simultaneously with the graphitic ${}^{13}$C NMR line shape function constrained such that its intensity ratio in the MRE relative to the FID was given by $G_{M}$ or G[δ]. The G[δ] constraint was implemented as a continuous function parameterized as an offset skew normal distribution according to a fit to experimentally derived G[δ] values. More details are given in the Supplementary Materials.

## Figures and Tables

**Table 1:** Mole fraction of graphitic carbon in poly(carbon monofluoride) samples determined by uncoupled ${}^{13}$C enhanced CPMG NMR at 16 ⅔ kHz MAS determined using two models for the gain function. The $G_{M}$ and G[δ] functions are given in the Supplementary Materials.

Sample	$x_{\mathrm{gr}}$/mol% ($G_{M}$)	$x_{\mathrm{gr}}$/mol% (G[δ])
(CF)${}_{n}$_gr-PC	48.5±3.6	45.7±2.7
(CF)${}_{n}$-CB	4.31±0.33	4.09±0.40
(CF)${}_{n}$-CF	0.70±0.15	0.72±0.43
(CF)${}_{n}$-PC	<0.05	

## Supplementary Materials

The following are available online at https://www.mdpi.com/1422-0067/21/11/3938/s1.

## Abbreviations

The following abbreviations are used in this manuscript:

NMR	nuclear magnetic resonance
CPMG	Carr-Purcell-Meiboom-Gill
XRD	X-ray diffraction
XPS	X-ray photoelectron spectroscopy
SEM/EDS	scanning electron microscopy/energy dispersive X-ray spectrometry
MAS	magic-angle spinning
CP	cross-polarization
FID	free induction decay
UDEFT	Uniform Driven Equilibrium Fourier Transform
PIETA	phase incremented echo train acquisition
PC	petroleum coke
CB	carbon black
CF	carbon fiber
gr	graphitic carbon
pXRD	powder X-ray diffraction
S/N	signal-to-noise
MRE	matched reconstructed echo
rms	root mean square
dFT	discrete Fourier transform

## Appendix A. Optimized Quantitative Echo Train Reconstruction

An echo signal with enhanced S/N ratio is constructed by weighted summation of the *k* individual echoes. This is a simple procedure in the time domain. We assume the NMR signal component to be enhanced by CPMG, S, has the same (suitably phased) functional form $s_{0}\left(t\right)$ with maximum response at t=0 in both the FID $\left(S_{0}\right)$ and the $j^{\mathrm{th}}$ echo ($S_{j}$), so that

(A1) $$\begin{array}{cc} S_{0}\left(t\right) & =Bs_{0}\left(t\right), \end{array}$$

(A2) $$\begin{array}{cc} S_{j}\left(t\right) & =BE\left(j\right)s_{0}\left(t\right). \end{array}$$

E(j) is the CPMG envelope function and will usually be given by Equation (1). In quantitative NMR the signal intensity, *B*, is to be measured with respect to that of the other NMR signal components. The reconstructed echo signal, $S_{W}$, is created by weighted summation of the $S_{j}$,

(A3) $$S_{W}\left(t\right)=B\left[\sum_{j=l+1}^{k}h\left(j\right)E\left(j\right)\right]s_{0}\left(t\right),$$

where h(j) is the mathematically exact weighting function. We omit the first *l* echoes to ensure complete dephasing of undesirable (e.g., fluorinated) signal components. Usually, l=0.

The amplitude of the reconstructed echo relative to the FID has increased by the bracketed quantity; this is the desired scaling to determine *B* from a signal enhanced $S_{W}\left(t\right)$ function. Signal analysis in NMR is rarely carried out in the time domain, however. In carrying this analysis into the frequency domain two important subtleties must be noted. Discrete Fourier transform (dFT) of the signals in Equations (A1) and (A3) respectively yield

(A4) $$\begin{array}{cc} ℜ\left\{S_{0}\left(\nu \right)\right\} & =\frac{1}{2}BQ\left(N_{0}\right)A\left(\nu \right), \end{array}$$

(A5) $$\begin{array}{cc} ℜ\left\{S_{W}\left(\nu \right)\right\} & =BQ\left(N_{W}\right)\left[\sum_{j=l+1}^{k}h\left(j\right)E\left(j\right)\right]A\left(\nu \right). \end{array}$$

By taking the real part in Equations (A4) and (A5) we emphasize that with suitable phasing of the NMR signal we are analyzing the absorption mode signal, A(ν). The $\frac{1}{2}$ factor in Equation (A4) is present because the FID signal cuts off $s_{0}\left(t\right)$ for t<0. Whole echo signals do not cut off $s_{0}\left(t\right)$ in this way. Second, the normalization convention chosen for the dFT may introduce a further scaling, Q(N), that depends on the number of points *N* in the signal being transformed. Here, we consider $N_{0}$ points in the FID signal and $N_{W}$ points in the reconstructed echo signal. These factors do *not*, in general, cancel when calculating the enhancement of the signal amplitude in the frequency domain,

(A6) $$\frac{ℜ\left\{S_{W}\left(\nu \right)\right\}}{ℜ\left\{S_{0}\left(\nu \right)\right\}}=2\frac{Q(N_{W})}{Q(N_{0})}\left[\sum_{j=l+1}^{k}h\left(j\right)E\left(j\right)\right].$$

The dFT scaling Q(N) is easily overlooked but is *essential* for an accurate quantitative analysis of signal intensities when they are compared separately but not independently, as in our method. Consider, for example, the pulse sequence in Figure 2 where *N* points are recorded over the period τ. Then the reconstructed echo has $N_{W}=2N$ points and the FID has $N_{0}=j_{0}N$ points, where $j_{0}=\tau_{0}/\tau$. Using the common normalization convention Q(N)=1/N, the relative dFT scaling is $Q\left(2N\right)/Q\left(j_{0}N\right)=j_{0}/2$. For another common convention, $Q\left(N\right)=1/\sqrt{N}$, we obtain a different result corresponding to $Q\left(2N\right)/Q\left(j_{0}N\right)=\sqrt{j_{0}/2}$. Only when $j_{0}=2$; that is, only when the FID and echo signals have the same number of points, is this scaling equal to unity independent of convention. Note that even for $\tau_{0}=\tau$, as in conventional CPMG, we have $j_{0}\neq 2$.

If unknown, the Q(N) factor used by the processing software being used can be determined by analyzing the amplitude of suitable test signals (e.g., constant or Gaussian functions) before and after dFT. If increasing the correlation between neighboring points is acceptable to the analysis, the need to know Q(N) can be circumvented by time domain zero filling of the FID and MRE signals so that they have the same number of points.

Noise is treated similarly. For any given point at time *t* we assume white noise given by $P\left[0,\sigma^{2}\right]\left(t\right)$, a probability function which generates a point with random amplitude distributed normally about zero with variance $\sigma^{2}$. We write for the time domain noise functions

(A7) $$\begin{array}{cc} N_{0}\left(t\right) & =P_{0}\left[0,\sigma^{2}\right]\left(t\right), \end{array}$$

(A8) $$\begin{array}{cc} N_{j}\left(t\right) & =P_{j}\left[0,\sigma^{2}\right]\left(t\right), \end{array}$$

for the FID and $j^{\mathrm{th}}$ echo respectively. After weighted reconstruction and transformation into the frequency domain it can be shown that

(A9) $$\begin{array}{cc} N_{0}\left(\nu \right) & =Q\left(N_{0}\right)\sqrt{N_{0}}\left(P_{0}\left[0,\sigma^{2}\right]\left(\nu \right)\right), \end{array}$$

(A10) $$\begin{array}{cc} N_{W}\left(\nu \right) & =Q\left(N_{W}\right)\sqrt{N_{W}\sum_{j=l+1}^{k}{\left[h(j)\right]}^{2}}\left(P_{W}\left[0,\sigma^{2}\right]\left(\nu \right)\right). \end{array}$$

The weighting function underneath the square root is squared because the intensity of the noise is measured by its standard deviation, whereas it is the variance of the noise that is additive. Therefore the enhancement of the noise level by echo reconstruction, measured by the ratio of the standard deviations in the frequency domain, is therefore

(A11) $$\frac{\sigma \left(N_{W}\right)}{\sigma \left(N_{0}\right)}=\frac{Q(N_{W})}{Q(N_{0})}\sqrt{\frac{N_{W}}{N_{0}}\sum_{j=l+1}^{k}{\left[h(j)\right]}^{2}}.$$

Both signal and noise functions are affected by the dFT scaling in the same way. By analyzing signals that are normalized with respect to the same level of noise, we obtain results that are independent of dFT normalization convention. This leads us to define the gain function,

(A12) $$G\left(k\right)=\frac{ℜ\left\{S_{W}\left(\nu \right)\right\}/\sigma \left(N_{W}\right)}{ℜ\left\{S_{0}\left(\nu \right)\right\}/\sigma \left(N_{0}\right)}=2\sqrt{\frac{N_{0}}{N_{W}}}\frac{\sum_{j=l+1}^{k}h\left(j\right)E\left(j\right)}{\sqrt{\sum_{j=l+1}^{k}{\left[h(j)\right]}^{2}}}.$$
