# Supplementary material for: Quantification of Uncoupled Spin Domains in Spin-Abundant Disordered Solids
Source: Int J Mol Sci. 2020 May 30;21(11):3938. doi: 10.3390/ijms21113938 (PMC7313085; doi:10.3390/ijms21113938)
Supplement: Supplementary file 1 [file ijms-21-03938-s001.pdf]

Supplementary Materials: Quantification of uncoupled spin domains in spin-abundant disordered solids

Brennan J. Walder, Todd M. Alam

The Supplementary Materials package includes

- A text file describing the supplementary file contents,
- the NMR pulse sequence (Bruker format),
- raw NMR data,
- *gnuplot* scripts and files used in the data analysis,
- this supplementary text.

Contents

1 Dependence of $S_M(k)$ on auxiliary variables **2**

2 CP MAS spectrum of (CF)$_n$_gr-PC **4**

3 Determination of $G(k)$ factors **5**

4 Envelope functions **7**

5 Other properties of ^{13}C relaxation **8**
5.1 Longitudinal relaxation 8
5.2 Dependence of relaxation MAS rate and recycle delay 9

References **10**

1. Dependence of $S_M(k)$ on auxiliary variables

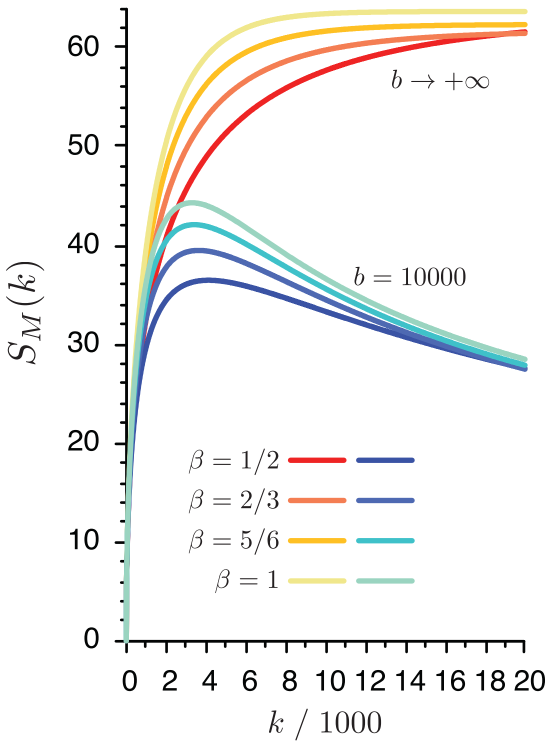

Figure S1. Dependence of Equation (4), where $G(k)$ is given by Equation (6), on the stretching exponent β when plotted against the number of echoes acquired, k. The two branches are distinguished by the values of b shown in the plot. The values $a = 8100$, $l = 0$, and $j_0 = 1$ are common to both branches.

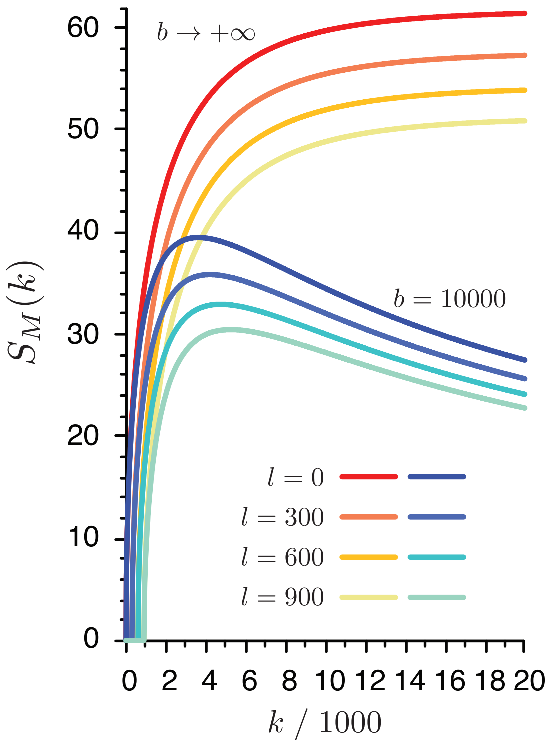

Figure S2. Dependence of Equation (4), where $G(k)$ is given by Equation (6), on the omission of the first l echoes when plotted against the number of echoes acquired, k. The two branches are distinguished by the values of b shown in the plot. The values $a = 8100$, $\beta = \frac{2}{3}$, and $j_0 = 1$ are common to both branches.

2. CP MAS spectrum of $(CF)_n$_gr-PC

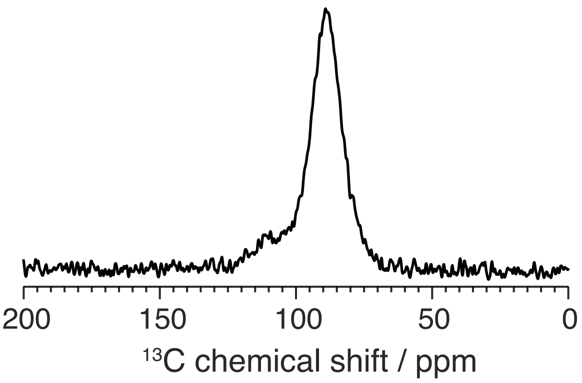

Figure S3. CP echo MAS ^{13}C NMR spectrum of $(CF)_n$_gr-PC acquired at 33 ⅓ kHz MAS. A 90% → 100% amplitude ramp was used with a short ^{19}F contact pulse of 0.05 ms with maximum rf amplitude of 83 kHz. The rf amplitude of the ^{13}C contact pulse was held constant at 46 kHz. The rf amplitude of the initial ^{19}F excitation pulse was 80 kHz. SPINAL-64 decoupling with a pulse element of 4.7 μs and an rf amplitude of 120 kHz was used during acquisition of the ^{13}C signal. A recycle delay of 2 s and 43008 scans led to an experiment time of 24.04 h. A $2\tau_R$ echo shift after CP was used to eliminate receiver dead time. Use of the short contact pulse selects for those ^{13}C that are directly bonded to ^{19}F.

3. Determination of $G(k)$ factors

Significant differences in the envelope function parameters T_{CPMG} and β are observed across the graphitic echo line shape, making the true envelope function, $E(j)[\delta]$, chemical shift dependent. These results are summarized in Table S1. Because of this differential relaxation the gain function $G(k)$ varies as a function of chemical shift. This is also shown in Table S1 and are plotted in Figure S4(a). The matched gain G using Equation (6) for each point is also reported. Neither zero filling nor other forms of apodization were used to prevent additional correlation of information between points. Reduced χ^2 values for the fit at each independent chemical shift point varied between 1.007 and 1.036, validating the stretch exponential model. Therefore, the resulting confidence intervals figured for G by propagation of error reliably reflect our certainty at each chemical shift.

The low x_{gr} of the fully fluorinated samples prevented an accurate determination of their envelope functions (see next section). For this reason, their relaxation properties were assumed to be the same as for $(CF)_n$_gr-PC, making the latter a reference sample for $E(j)$. The $G[\delta]$ functions used in the analysis of $(CF)_n$-CB ($j_0 = 6$) and $(CF)_n$-CF ($j_0 = 8$) were simply derived from the $(CF)_n$_gr-PC data by adjusting for the different values of j_0 and fitting to an offset skew normal distribution. The curve for $(CF)_n$-CF is also shifted by -16.6 ppm (two points) to compensate for the change in chemical shift we observe for $\mathcal{S}_W(\nu)$ of $(CF)_n$-CF relative to $(CF)_n$-CF and $(CF)_n$_gr-PC. This is illustrated in Figure S4(b). Note the change in the maxima of $G[\delta]$ for the other samples is driven purely by j_0 and relates to the additional noise in the FID introduced by the longer τ_0 used for the fully fluorinated samples.

For comparison, we also provide the fully matched $G_M(30000)[\delta] \equiv G_M[\delta]$ values for each point Table S1. This would be the result if a chemical shift dependent weighting function $h(j)[\delta]$ was chosen to match $E(j)[\delta]$ at each point. We see that difference between $G[\delta]$ and $G_M[\delta]$ is most significant near the edges of the line shape. Since the signal is weakest here there is little benefit in implementing a chemical shift dependent weighting function.

Table S1. Variation of the CPMG envelope function parameters as a function of chemical shift for $(CF)_n$_gr-PC ($k = 30000$, $j_0 = 4$). The third column gives the corresponding gain $G[\delta]$ where the same $h(j)$, based upon the best fit envelope function for the integrated line shape (final row), is used to calculate each entry. The value of $G_M[\delta]$ is calculated using Equation (6), as would result if a chemical shift dependent weighting function $h(j)[\delta]$ was chosen. The error range reported for T_{CPMG} and β are the 1σ confidence intervals about the best fit reported by the fitting program. The error range for G is calculated from the other ranges by propagation of error.

Chemical shift	T_{CPMG} / s	β	$G[\delta]$	$G_M[\delta]$
165.1 ppm	1.933 ± 0.154	0.689 ± 0.035	57.51 ± 3.93	77.65 ± 3.11
156.8 ppm	2.849 ± 0.148	0.628 ± 0.018	80.75 ± 3.19	94.46 ± 2.46
148.5 ppm	4.460 ± 0.123	0.642 ± 0.011	108.87 ± 2.02	118.07 ± 1.62
140.2 ppm	7.157 ± 0.110	0.629 ± 0.006	145.45 ± 1.24	148.86 ± 1.09
132.0 ppm	11.389 ± 0.094	0.638 ± 0.004	184.32 ± 0.71	184.78 ± 0.69
123.7 ppm	16.759 ± 0.085	0.641 ± 0.003	217.78 ± 0.47	217.98 ± 0.47
115.4 ppm	20.606 ± 0.084	0.633 ± 0.003	234.71 ± 0.40	235.71 ± 0.41
107.1 ppm	20.923 ± 0.095	0.626 ± 0.003	235.45 ± 0.45	236.57 ± 0.46
98.8 ppm	17.328 ± 0.124	0.619 ± 0.004	219.34 ± 0.65	219.68 ± 0.66
90.5 ppm	11.795 ± 0.175	0.594 ± 0.006	186.42 ± 1.22	186.53 ± 1.20
82.3 ppm	6.630 ± 0.223	0.566 ± 0.011	141.28 ± 2.53	143.69 ± 2.26
74.0 ppm	4.005 ± 0.272	0.569 ± 0.020	105.64 ± 4.62	112.65 ± 3.78
65.7 ppm	2.899 ± 0.325	0.637 ± 0.040	81.27 ± 6.95	95.24 ± 5.35
Integrated	12.769 ± 0.048	0.571 ± 0.002	192.19 ± 0.30	

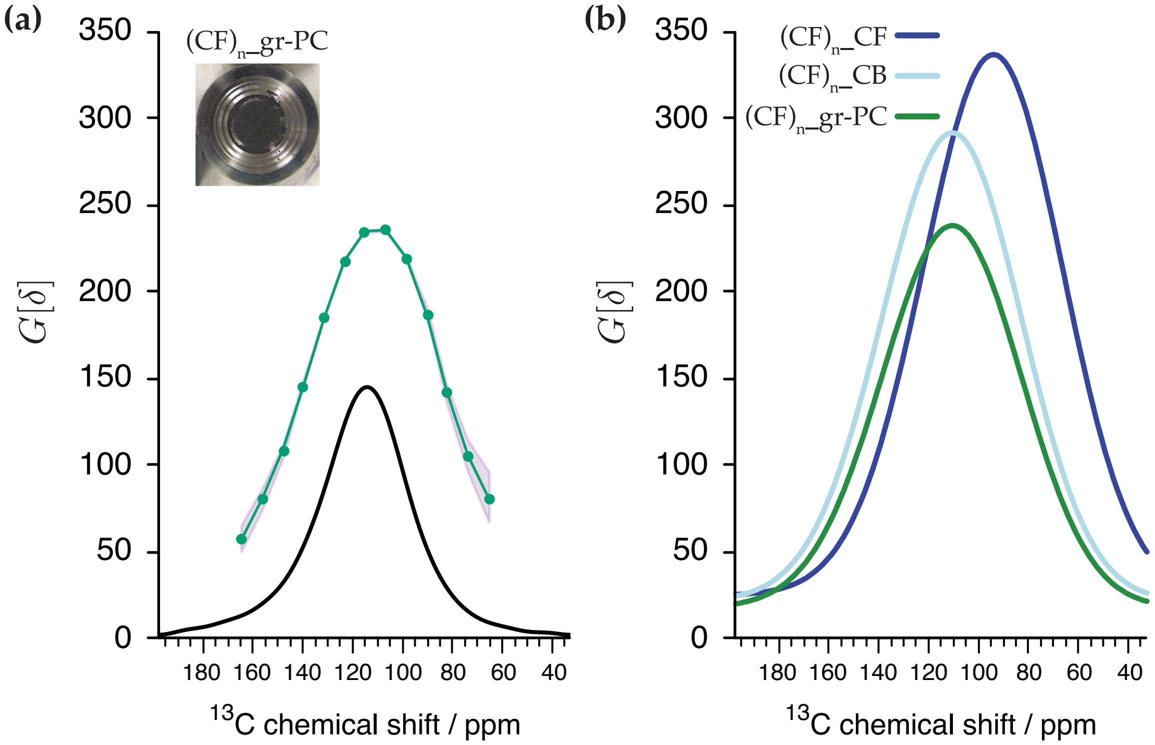

Figure S4. Chemical shift dependence of the gain functions used in the analysis of the experimental data. **(a)** Graph of the G values given in Table S1 (green) for $(CF)_n$_gr-PC ($k = 30000$, $j_0 = 4$). The shaded band represents the 2σ confidence interval obtained by propagation of error. The arbitrarily scaled graphitic ^{13}C NMR signal $\mathcal{S}_W(\nu)$ is overlaid in black for reference. **(b)** Continuous chemical shift dependent gain functions derived from the $(CF)_n$_gr-PC data and applied to the other samples after adjusting for j_0 and, for $(CF)_n$-CF, the chemical shift change observed in its $\mathcal{S}_W(\nu)$.

4. Envelope functions

Figure S5 graphs the integrated echo intensities and best fit envelope functions $E(j)$ (curves) for $(CF)_n$_gr-PC, $(CF)_n$-CB, and $(CF)_n$-CF. The best fit parameters characterizing the decay given in Table S2. Given the degree of uncertainty, the results are not different enough to render the decision to use $(CF)_n$_gr-PC as a reference for $E(j)$ unjustified, as discussed in the main text.

Table S2. Best fit parameters characterizing the envelope functions shown in Figure S5.

Sample	T_{CPMG} / s	β
$(CF)_n$_gr-PC	12.769 ± 0.048	0.571 ± 0.002
$(CF)_n$-CB	13.924 ± 0.821	0.599 ± 0.029
$(CF)_n$-CF	26.532 ± 3.762	0.57 (constrained)

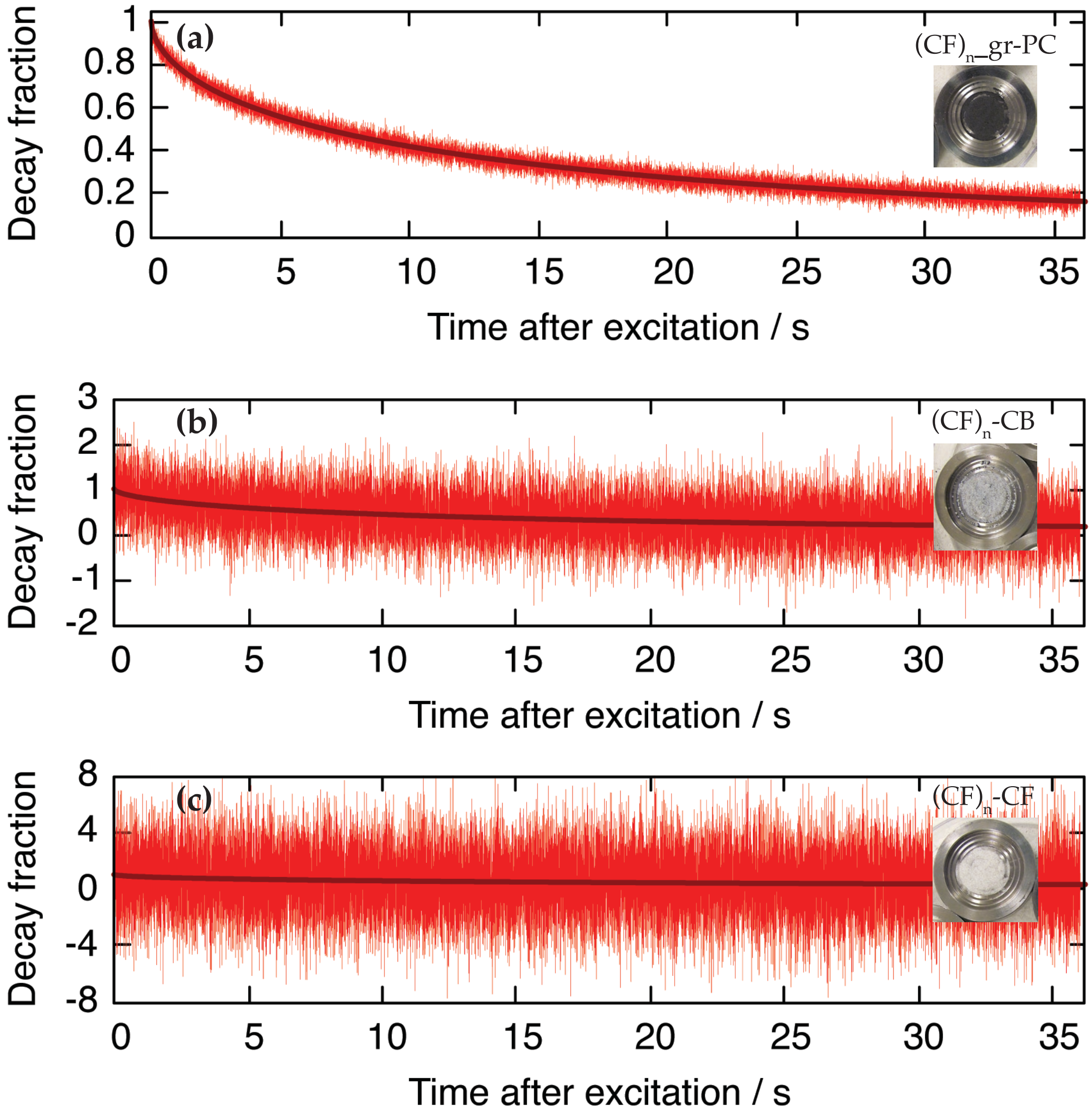

Figure S5. Integrated echo intensities and best fit envelope functions $E(j)$ for **(a)** $(CF)_n$_gr-PC, **(b)** $(CF)_n$-CB, **(c)** $(CF)_n$-CF.

5. Other properties of ^{13}C relaxation

5.1. Longitudinal relaxation

As is typical of dilute nuclei such as ^{13}C at its 1.11% natural abundance in disordered solids, the longitudinal recovery of magnetization is described well using a stretched exponential function [1],

$$\frac{M(\tau_{\rm rd})}{M_{\rm eq}} = 1 - e^{-(\tau_{\rm rd}/T_1^b)^{\beta_1}}, \tag{S1}$$

where the fraction of recovered magnetization $M/M_{\rm eq}$ as a function of the recycle delay between scans, $\tau_{\rm rd}$, is determined by the characteristic longitudinal relaxation time T_1^b and the corresponding stretching exponent β_1.

Saturation recovery experiments were used to measure longitudinal relaxation of ^{13}C in poly(carbon monofluoride). Measurements were performed selectively for graphitic domains using CPMG enhancement of $(CF)_n$_gr-PC. For fluorinated domains selective measurement was performed on $(CF)_n$-PC by using CP followed by a z-filter. The duration of the z-filter storage interval, τ_z, was increased and the longitudinal relaxation parameters were determined using

$$\frac{M(\tau_z)}{M_{\rm eq}} = e^{-(\tau_z/T_1^b)^{\beta_1}}. \tag{S2}$$

Data and fits to Equations (S1) or (S2) are shown in Figure S6. The parameters we determine for the two components of the ^{13}C NMR signal, corresponding to the graphitic and fluorinated domains, are given in Table S3. In order to recover 99% of the magnetization, the threshold conventionally considered necessary for NMR to be effectively quantitative, less than 5 min is required for the graphitic domains whereas over 10 min is required for the fluorinated domains. At an MAS rate of 16 ⅔ kHz we select $\tau_{\rm rd} = 12$ min for our experiments.

Table S3. Carbon-13 NMR relaxation parameters measured on samples of poly(carbon monofluoride) spinning under compressed air. Fluorinated domain parameters were measured on $(CF)_n$-PC. Graphitic domain parameters were measured on $(CF)_n$_gr-PC. The $T_{\rm CPMG}$ and β parameters are analyzed as the integral over the graphitic carbon signal in a CPMG experiment having $\tau = \tau_0 = 2.4$ ms ($j_0 = 1$) and $k = 3200$ echoes. The range establishes the 1σ confidence interval.

Domain	MAS rate	T_1^b / s	β_1	$\tau_{\rm rd}$ / s	$T_{\rm CPMG}$ / s	β
Graphitic	16 ⅔ kHz	34.71 ± 0.56	0.661 ± 0.009	30	10.20 ± 0.18	0.630 ± 0.013
				300	13.90 ± 0.22	0.614 ± 0.015
	33 ⅓ kHz	29.78 ± 0.53	0.688 ± 0.010	30	9.72 ± 0.17	0.614 ± 0.013
				300	13.55 ± 0.22	0.611 ± 0.015
Fluorinated	16 ⅔ kHz	49.44 ± 2.78	0.696 ± 0.039			
	33 ⅓ kHz	82.66 ± 7.58	0.660 ± 0.059			

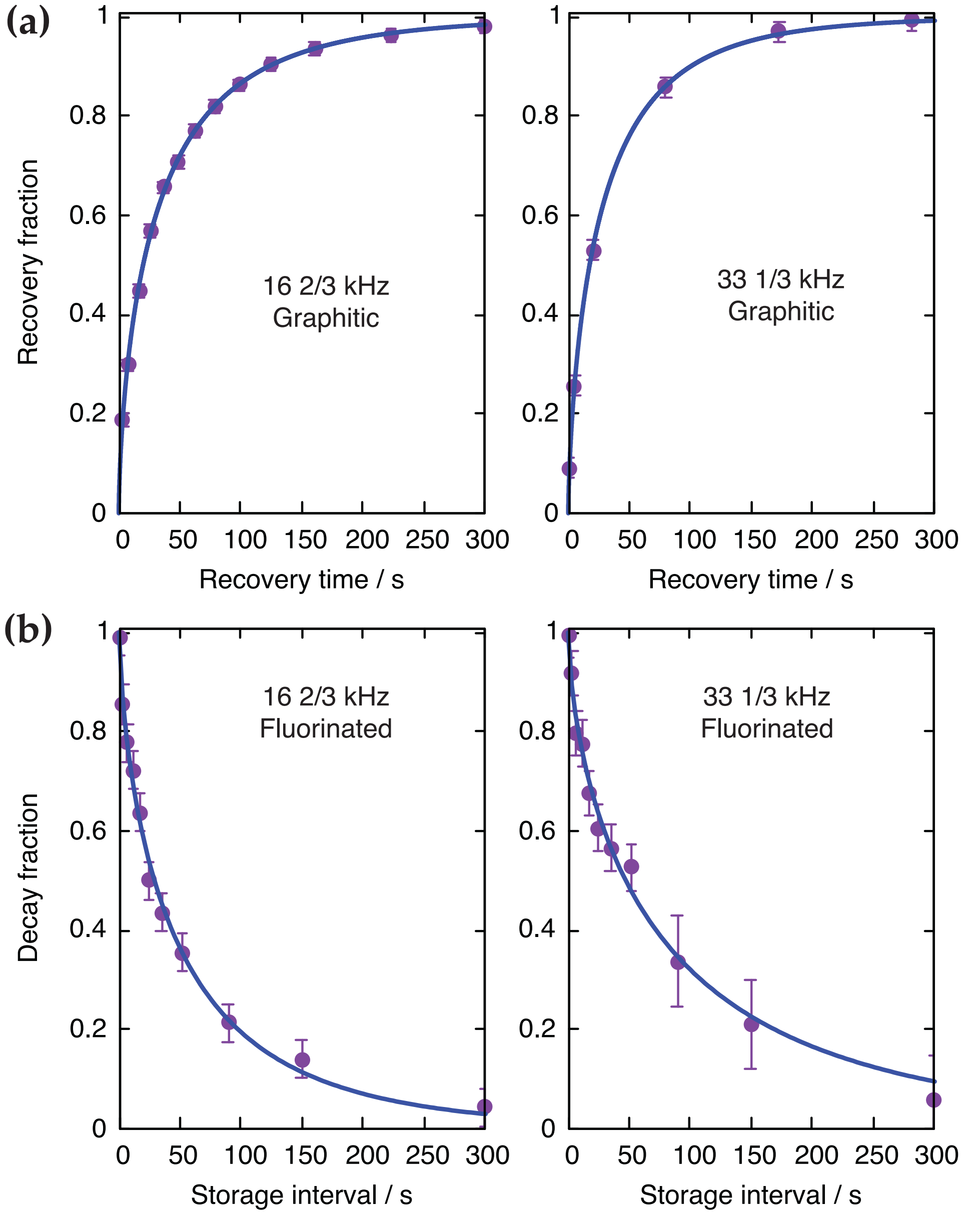

Figure S6. Characterization of ^{13}C longitudinal relaxation at different MAS rates for **(a)** the graphitic domains of $(CF)_n$_gr-PC, **(b)** the fluorinated domains of $(CF)_n$-PC. Several additional points up to 1200 s (16 ⅔ kHz) and 3600 s (33 ⅓ kHz), not plotted, were also collected to ensure complete recovery. Curves represent the best fit to Equations (S1) or (S2). The plotted error bars represent 2σ confidence intervals.

5.2. Dependence of relaxation MAS rate and recycle delay

In Table S3 we note the T_1^b of the fluorinated domains is significantly affected by the MAS rate, nearly doubling when the spin rate is doubled. The β_1 parameter also falls somewhat. This suggests that a substantial reduction of ^{19}F-driven ^{13}C spin diffusion [2,3], which transports ^{13}C magnetization to and from paramagnetic defects that are the dominant cause of relaxation [4,5], is responsible. Graphitic T_1^b is much less affected by the MAS rate, possibly because chemical shift dispersion plays a greater role than MAS rate in suppressing ^{13}C spin diffusion for these domains [2,6–8], which will already be much weaker as ^{19}F-driven processes are inoperative. Differences in sample temperature may also play a role.

Table S3 also provides data relaxed to the integrated envelope function for the graphitic regions of $(CF)_n$_gr-PC, acquired under different conditions than those reported in Table S1. Here, the

MAS rate and recycle delay is varied for otherwise identical CPMG experiments. Similar to the case of longitudinal relaxation, T_{CPMG} and β are not significantly affected by the MAS rate. In contrast, a substantial increase in T_{CPMG} is noted at the longer τ_{rd}. This indicates that the parameters characterizing $E(j)$ are coupled to the extent of longitudinal relaxation and is consistent with relaxation induced by paramagnetic centers, as a longer recycle delay permits a greater contribution by those ^{13}C nuclei farther away from paramagnetic defects thus having longer T_1 and T_2. The differences in the envelope functions are visualized in Figure S7.

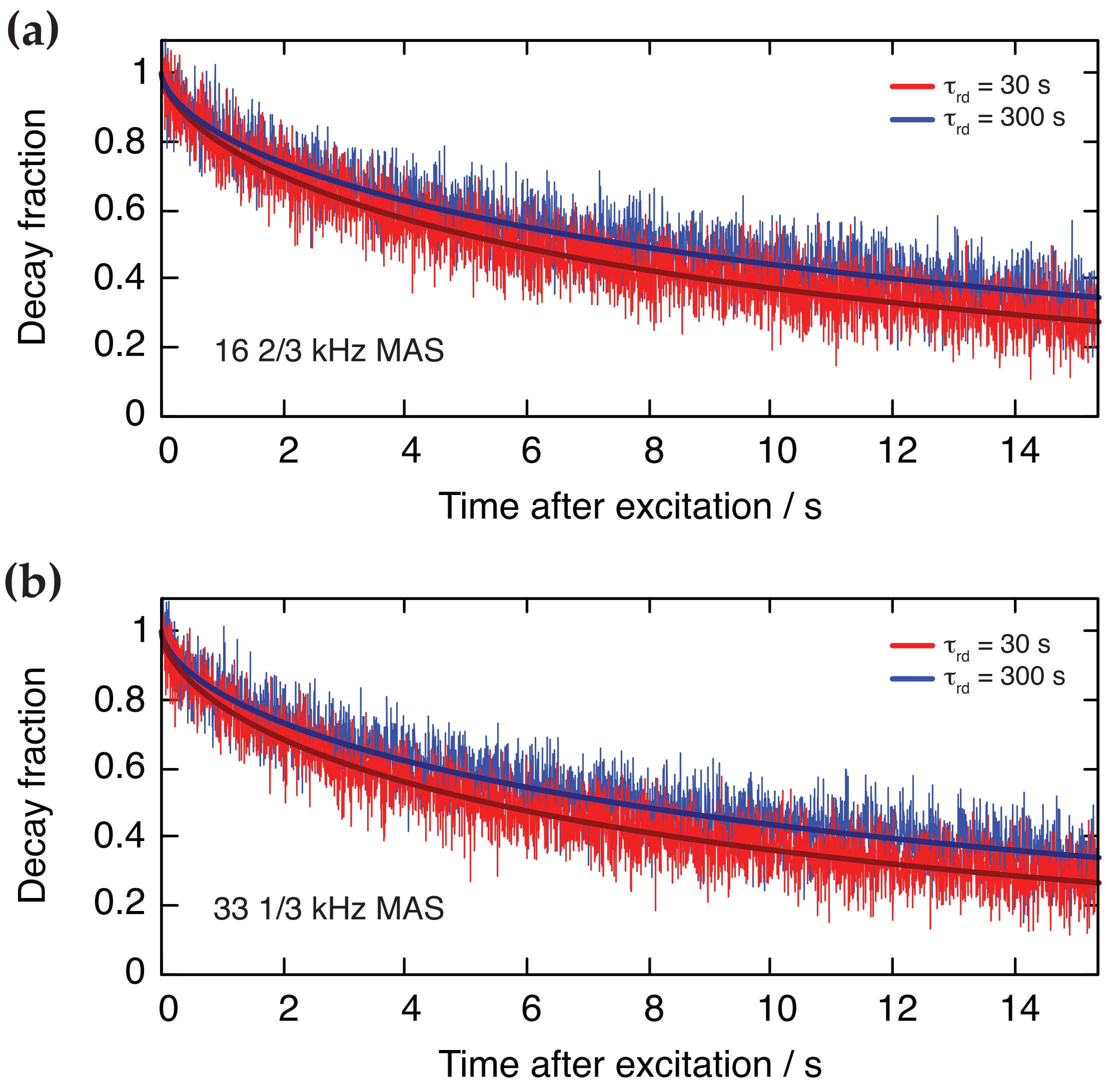

Figure S7. Integrated ^{13}C MAS NMR CPMG echo intensities and best fit envelope functions (curves) for $(CF)_n$_gr-PC at MAS rates of **(a)** 16 ⅔ kHz, **(b)** 33 ⅓ kHz, corresponding to the experiments with different recycle delays as described in Table S3.

References

1. Narayanan, A.; Hartman, J.S.; Bain, A.D. Characterizing Nonexponential Spin-Lattice Relaxation in Solid-State NMR by Fitting to the Stretched Exponential. *Journal of Magnetic Resonance, Series A* **1995**, *112*, 58–65. doi:10.1006/jmra.1995.1009.
2. Suter, D.; Ernst, R.R. Spin diffusion in resolved solid-state NMR spectra. *Physical Review B* **1985**, *32*, 5608–5627. doi:10.1103/PhysRevB.32.5608.
3. Levitt, M.H.; Raleigh, D.P.; Creuzet, F.; Griffin, R.G. Theory and simulations of homonuclear spin pair systems in rotating solids. *The Journal of Chemical Physics* **1990**, *92*, 6347–6364. doi:10.1063/1.458314.

4. Bloembergen, N. On the interaction of nuclear spins in a crystalline lattice. *Physica* **1949**, *15*, 386–426. doi:10.1016/0031-8914(49)90114-7.
5. Lowe, I.J.; Tse, D. Nuclear Spin-Lattice Relaxation via Paramagnetic Centers. *Phys. Rev.* **1968**, *166*, 279–291.
6. Maricq, M.M.; Waugh, J.S. NMR in rotating solids. *J. Chem. Phys.* **1979**, *70*, 3300–3316.
7. Clough, S.; Gray, K.W. The Stochastic Theory of the Nuclear Magnetic Resonance Line in Rotating Solids. *Proceedings of the Physical Society* **1962**, *79*, 457. doi:10.1088/0370-1328/79/3/301.
8. Clough, S.; Gray, K.W. Spin Diffusion and Nuclear Magnetic Resonance in Rotating Solids. *Proceedings of the Physical Society* **1962**, *80*, 1382. doi:10.1088/0370-1328/80/6/120.
